# Influence of Fiber Content and Dosing Position on the the Mechanical Properties of Short-Carbon-Fiber Polypropylene Compounds

**DOI:** 10.3390/polym14224877

**Published:** 2022-11-12

**Authors:** Thomas Höftberger, Florian Dietrich, Gernot Zitzenbacher, Christoph Burgstaller

**Affiliations:** 1 Transfercenter für Kunststofftechnik GmbH, Franz-Fritsch-Str. 11, 4600 Wels, Austria; 2 School of Engineering, University of Applied Sciences Upper Austria, Stelzhamerstraße 23, 4600 Wels, Austria

**Keywords:** fiber length, mechanical properties, twin-screw compounding

## Abstract

The properties of short-fiber-reinforced composites depend on the fiber length of the reinforcing fibers. This fiber length is typically influenced by processing to different extents. In this work, we investigate the influence of processing, i.e., the influence of residence time achieved via different dosing points in compounding, and the fiber content on the fiber length and mechanical properties of short-carbon-fiber-reinforced polypropylene. We found that, with increasing fiber content, the fiber length decreases from 900 to 300 µm after compounding and from 500 to 250 µm after injection molding. Additionally, a decrease in residence time in the compounder leads to an increase in the fiber length of approx. 300 µm compared to the longer residence time. This is later reduced by the injection molding step, but the longer fibers are still longer in the final molded test specimen, thus resulting in a 5–10% increased tensile strength and elastic modulus as well as an some increase in impact strength. As the injection molding step showed considerable fiber length reduction (down to 250 µm), further investigations of injection molding should be undertaken to preserve fiber length better for the increased performance of these composites.

## 1. Introduction

The use of fiber-reinforced composites is continuously growing due to high demand for structural and lightweight composites as well as high performance production processes in many industrial sectors. Not only fiber length, but also matrix type, fiber volume content, fiber orientation and interfacial adhesion correlate directly with composite properties [1,2,3]. Long-fiber-reinforced plastics offer high mechanical performance for a wide range of applications, but they are still complicated and expensive to produce, requiring a high level of automation and complex processes. On the contrary, short fiber composites are mixed in one or more melt processing steps using heat and shear energy for mixing and adhesion between the fiber and matrix to produce a granulate. These pellets can then be injection-molded into parts or extruded to create profiles, to mention a few examples.

There are a variety of possible reinforcing fibers, both natural and synthetic. Most natural fibers have a cellulose base and grow as reinforcing fibers in plants. Another natural fiber type are animal fibers, which have protein as a base [4]. As temperatures above 200 °C are often necessary in plastic processing, either due to the polymer melting point itself or to reduce the viscosity in processing by raising the temperature, which is often the case for polypropylene, the low thermal stability of natural fiber reinforcements is a disadvantage of both fibers [5].

As for synthetic fibers, a distinction can be made between inorganic and organic fibers. The latter, such as polyester or polyamide, are not often used as reinforcing fibers due to their anisotropic behavior in the longitudinal direction of the fiber. These fibers yield their mechanical properties due to drawing stages, where the polymer chains are oriented and therefore the longitudinal properties are increased. With applying heat in the different polymer processing steps, these orientations often also relax to a more unordered state, thus reducing the mechanical properties; therefore, these fibers are more commonly used in the textile industry and are rare in thermoplastic composites. Another organic fiber group are the manmade cellulose-based ones, which have some reinforcement application, for example, in pneumatic tires [6]. The applications of such fibers always have to keep in mind the aforementioned limited thermal stability. The last group of synthetic fibers are inorganic fibers, such as carbon or glass. While glass fibers have a good balance between their relative high properties and reasonable costs, and are the most prevalent, the properties of carbon fibers exceed those of nearly all other fibers [7]. Carbon fibers tolerate thermal stress during melt processing well but suffer from high shear stress and, therefore, are prone to breaking into shorter fragments. Compared to glass fibers, carbon fibers are far more sensitive to shear stress in terms of fiber breakage. The main reason for investigating and improving the existing production processes is to maintain the fiber length of short-fiber-reinforced plastic compounds throughout the manufacturing process, as fiber length is a major influence on the composite properties of the finished parts [8,9,10].

Fiber degradation mechanisms have been investigated in many scientific publications [11,12,13,14,15,16]. An early study shows that fiber breakage occurs mostly during the early process of fiber dispersion in the compounding, i.e., melt mixing, process [14]. Another investigation shows the fiber shortening along the extruder barrel [15], but also fiber shortening, were observed when processing the fibers with an internal mixer, as only the longer fibers improved in the composite properties [16]. Fiber breakage occurs not only in the melting zone, but also fibers added afterwards experience sufficient shear forces to fracture [12]. Furthermore, processing parameters, such as screw design, screw speed, temperature profile and feed rate, influence the final fiber length, mainly due to the variations in the overall shear forces, which are applied to the material in the process [17,18,19,20,21,22,23]. Another important influence is the fiber content itself. Different studies show a higher fiber content leads to shorter fibers due to increased fiber–fiber interactions [8,15,24,25].

Most of these studies refer to glass fibers, but short-carbon-fiber composites are of great interest in lightweight applications, if the expected performance can hold up to the high expectations of their theoretically achievable properties. Only a few publications have dealt specifically with the topic of processing carbon fibers. In one work, an internal mixer (and a milling step for granuling) was used to produce composites with different fiber lengths. The longer fibers showed improved properties for modulus and tensile strength, for example [26]. A higher fiber content, leading to higher fiber degradation, was reported in another study, where carbon fibers were mixed with an internal mixer into PMMA [27]. Another work used a micro-compounder for mixing the carbon fibers, and also found correlations between the fiber length and the reinforcing properties of the carbon fibers [28]. Nevertheless, we wanted to investigate the effect of a co-rotating twin screw extruder on short-carbon-fiber composites, as these processing machines are state of the art for high-volume compound production. Therefore, the aim of this work is to apply the knowledge gained in short-glass-fiber-reinforced plastics to short-carbon-fiber-reinforced polypropylene, focusing on the influence of fiber weight fraction and processing length (varied via the dosing location) in melt processing, i.e., compounding the fibers with a co-rotating twin screw extruder, on fiber length and composite properties.

These investigations are carried out to show the fiber lengths can be achieved after the compounding step and what differences can be achieved in fiber length in comparison to the standard processing. Furthermore, we wanted to show if higher fiber lengths are beneficial for the composite properties in the final part, i.e., the injection-molded universal test specimen.

## 2. Materials and Methods

The matrix material used for this work was a polypropylene (PP) homopolymer HE125MO (supplied by *Borealis*, Linz, Austria), which exhibits an MFR-value of about 12 g/10 min (at 230 °C and 2.16 kg piston weight), a density of 0.91 g/cm^3^ and a tensile modulus of about 1550 MPa. The material was used because of its good general processability in compounding and injection molding. For the fiber reinforcement, short-cut carbon fibers (CF), supplied by *Zoltek*, Nyergesujfalu, Hungary (PX35-55) with approx. 7 µm diameter, a density of 1.8 g/cm^3^, a PP-compatible sizing and 6 mm length, were used. As a coupling agent, Scona TPPP 8112 GA (supplied by *BYK*, Schkopau, Germany), which is a maleic anhydride grafted polypropylene with a graft level of about 1.4 wt% maleic anhydride and exhibiting an MFR value of about 80 g/10 min (at 190 °C and 2.16 kg piston weight), was used to improve the interaction between the fibers and matrix.

From these materials, multiple compositions were produced, as seen in Table 1, using a co-rotating twin screw extruder (ThermoPrism TSE 24HC, Thermo Fisher Scientific, Karlsruhe, Germany) with 24 mm screw diameter and a processing length of 40 L/D. All three components were dosed using a gravimetric dosing system. PP and coupling agent were dosed into the intake of the extruder, and fibers were dosed either in Zone 3 (equal to 28 L/D residual processing length) or Zone 8 (equal to 8 L/D residual processing length) via a side feeder unit. The throughput was kept constant at 10 kg/h with a screw rotational speed of 300 min^−1^. The extruded melt strands were cooled by water bath, cut to 5 mm long granules by means of a strand cutter and dried at a temperature of 90 °C for at least 1.5 h. The temperature profile used in compounding as well as the description of the screw geometry can be seen in Table 2. From the produced granules, universal test specimens (specimen geometry in accordance with ISO 527) were injection-molded (Victory 80/330, Engel, Schwertberg, Austria with a 35 mm screw diameter and a maximum clamping force of 80 t) following ISO 3167. The processing parameters were kept the same for all mixtures (Table 3). The test specimens were stored for at least 88 h under standardized conditions (23 °C and 50 % relative humidity) before any further testing.

With these specimens, material characterization was performed. Tensile testing was carried out according to ISO 527 on a Z020 universal test machine (Zwick/Roell, Ulm, Germany), equipped with pneumatic clamps with metal inserts with pyramid serrations, with a crosshead speed of 1 mm/min for determining the elastic modulus and 5 mm/min until the break of the samples. Universal test specimens according to ISO 527 were used. Three replicates were tested for each mixture. Charpy impact properties were tested using a Zwick/Roell 5113.300 pendulum impactor (Zwick/Roell, Ulm, Germany) according to ISO 179 on notched and unnotched samples. The specimens were punched out from the parallel part of the universal test specimen, yielding a prismatic specimen with 80 × 10 × 4 mm^3^ with five replicates for each material and impact test. Density was measured according to ISO 1183-1 using a density kit (YDK01) and a scale (M-pact AX224), both from Sartorius, Göttingen, Germany. As specimens, prismatic specimens with approx. 40 × 10 × 4 mm^3^ (halves of the specimen after notched impact testing) were used, where for each mixture three replicates were tested.

To determine the fiber weight fracture in the different compounds, a macro thermogravimetric analysis was performed using a Makro-TGA 701 device from Leco, Mönchengladbach, Germany. The same specimens as for density measurements were used, and three replicates were tested for each formulation. Determining density and fiber weight fraction on the same type of specimen (as well as knowing the fiber density from the data sheet) enables the calculation of the fiber volume fraction (by dividing the weight fraction of the fiber by the fiber density and multiply this with composite density).

**Figure 1:**
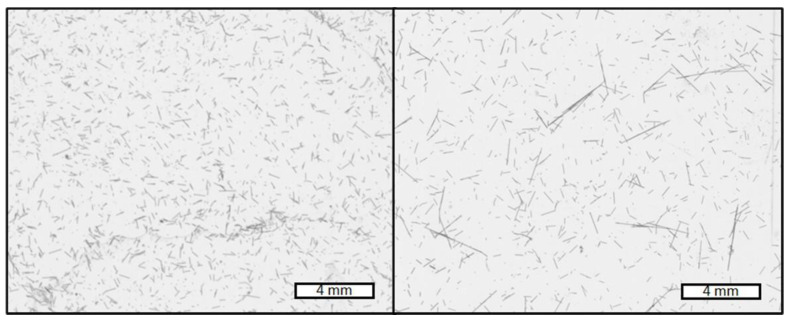
Example of images generated by scanning the Petri dish with the fibers dispersed in water for evaluating the fiber length using the Fasep-software. Fibers from samples with 15 wt% of carbon fibers, dosed into zone 3 (**left**) and zone 8 (**right**). The scale bar represents 4 mm length. The reader can see a qualitative difference in how the fibers are broken down: in the right picture with a shorter processing length, several longer fibers can be seen in comparison with only shorter fibers in the left picture, where the longer processing length was applied to the compound.

Fiber length measurements were performed using FASEP image analysis software (IDM Systems, Darmstadt, Germany). A piece of the parallel part of universal test specimen was treated in an oven at a temperature of 450 °C for 3 h to burn away the polymer, therefore yielding the carbon fibers. They were then dispersed in deionized water (about 2 mg of fibers in 100 mL). Afterwards, a Petri dish was filled with this solution and an image was taken using an Epson-Perfection V800 scanner. This image was then processed using the FASEP software to evaluate the fiber length. An example for such images is shown in Figure 1. In total, 3000 to 4500 fibers per formulation were measured.

The analysis started by arranging the fiber lengths in classes of 112 µm (ranging from 0 to 9000 µm). Then, the length weighted fiber lengths were calculated as shown in Equation (1) by summing the products of the number of fibers in each class with the class length average squared and dividing this by the sum of the products of the number of fibers in each class with the class length average.

(1) $$l_{\mathrm{lw}}=\frac{\sum n_{i}{l_{i}}^{2}}{\sum n_{i}l_{i}}$$

where l_lw_ is the length weighted fiber length, n_i_ is the number of fibers in each class and l_i_ is the class length average.

## 3. Results and Discussion

**Figure 2:**
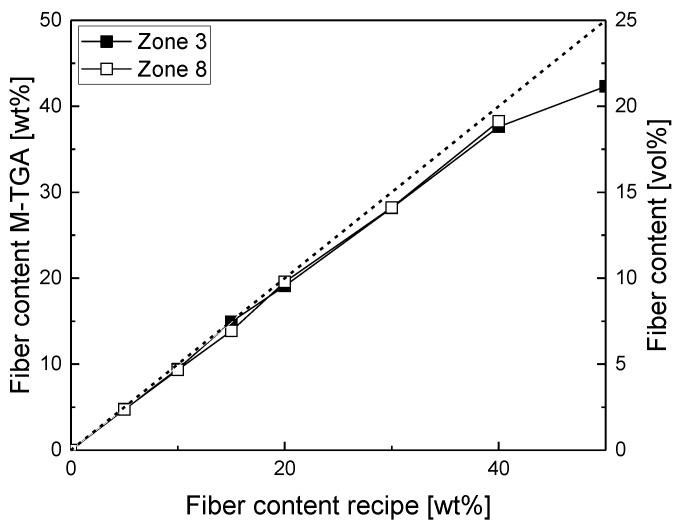
Comparison of the fiber content determined with the M-TGA on universal test specimen vs. the nominal fiber content of the recipes for two different dosing positions (zone 3 and 8) in compounding (fiber content in vol% is shown on the secondary *y*-axis as an orientation, as the following properties are presented in correlation with it instead of wt%).

As fiber content is one of the main influencing factors in composites, it is necessary to check whether the setpoint of the dosing and the actual fiber content are in good agreement. Therefore, we compared the setpoint as well as the results from the Makro-TGA measurements in Figure 2. It shows that the determined fiber content is equal to the fiber content in the recipe up to 20 wt%, but with higher fiber contents, the determined fiber content is lower than the dosed one, and this difference increases with increasing fiber content. The reason for this difference is most likely that, with increasing the fiber volume, the dosing becomes more difficult due to the light weight and, therefore, the high volume of the fibers. This issue is true for both the dosing hopper feeding into the sidefeeder and the sidefeeder pushing the fibers into the melt.

Another observation we made here is that the melt strands of the materials, where the fibers were dosed in the later position on the extruder and were harder to haul off due to swelling of the strands, most likely due to entrapped air that could not be degassed because of the late dosing point, but also due to some lack of wetting out all the single carbon fibers with the matrix. This swelling led to issues in pulling off the strands, as these tend to tear easily, up to a point where the production of the strand was not possible anymore at 50 wt% of fibers when dosed in zone 8. When dosing the fibers in zone 3, the strands showed an overall better behavior, but in high fiber contents, some porosity could still be seen. This shows that a higher process length can improve the quality by reducing the air entrapped in the compound, but cannot completely degas it.

**Figure 3:**
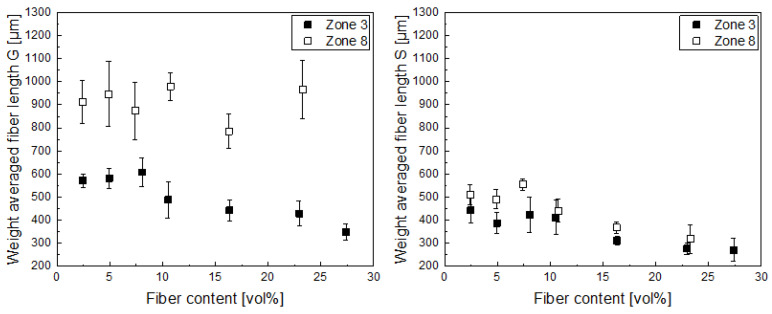
The weight average fiber length over the fiber volume content of the granules (G, (**left**)) and the universal test specimen (S, (**right**)) for carbon-fiber polypropylene composites produced with two different dosing positions in compounding (zone 3 and 8).

In the next step, the fiber length was investigated in the produced granules (G) as well as in the test specimen (S), as shown in Figure 3. We choose to show the weight average fiber length as opposed to a number average fiber length, as the latter would over-represent smaller fiber lengths, especially taking into account that the composite properties depend on the volumetric proportions of the fibers in the matrix. Additionally, from this point onwards, we present the fiber fraction in all figures in volume percent, as the composite properties correlate with it instead of weight fractions. Looking at the fiber lengths in the granules, two effects can be seen. On the one hand, when the residence time is shorter (dosing in zone 8), the fiber length is preserved better than for the dosing with the higher residence time (zone 3). On the other hand, the fiber length decreases with increasing fiber content. This effect is seen well for the compounds with a longer residence time (zone 3), but does not seem to be true for those with a short residence time. We suspect the latter to be an effect of the very short mixing time, so the fiber–fiber interaction and the overall shear input was much lower, resulting in overall longer fibers, which are not fully dispersed in the matrix. Looking at the fiber lengths in the universal test specimen, it is evident that fiber lengths are further reduced due to the additional processing step. Here, the length reduction is more pronounced for the compounds where the fibers were dosed later in the compounding process (zone 8), as in the injection-molding step adds additional shear forces, therefore distributing the fibers better, but also breaking them. Nevertheless, the residual fiber lengths achieved are still higher than the ones achieved when processing the fibers in compounding with a longer residence time (dosing in zone 3). This also shows that there is still a considerable fiber length reduction in injection molding, although the processing parameters were chosen from our experience to preserve fiber length, i.e., with a low back pressure; nevertheless, the higher fiber length after compounding still yields longer fibers in the final composite.

**Figure 4:**
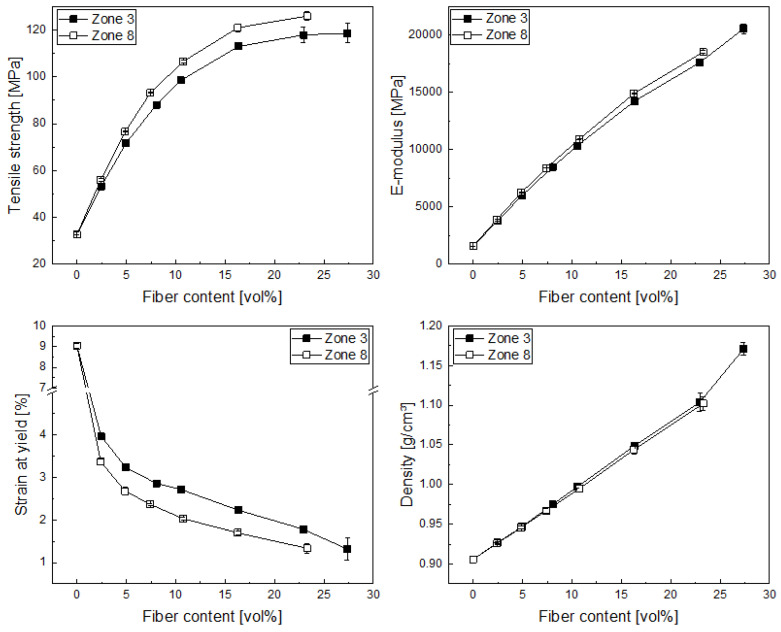
Tensile strength (**top left**), elastic modulus (**top right**), strain at yield (**bottom left**) and composite density (**bottom right**) for carbon-fiber polypropylene composites produced with two different dosing positions in compounding (zone 3 and 8).

The mechanical properties of the produced compounds were analyzed in terms of the influence of the fiber length. In general, all the specimens broke in the same fracture mode, i.e., there was no visible sign of delamination, but the composites broke completely after loading, and some fiber pullouts could be seen at the fracture surface, which was expected even for such short reinforcing fibers, as when the crack propagates, not all the fibers are aligned alike and therefore both fiber breakage and fiber pull out take place. In the case of the elastic modulus (Figure 4), an increase with increasing fiber volume content can be found, as the fibers exhibit a much higher modulus than the matrix. Some influence of the fiber length can be seen at higher fiber volume fractions, but these differences are in the region of below 5%. In the case of the tensile strength, we also found an increase with increasing fiber volume content, which flattens at higher volume fractions, most probably due to fiber length reduction and increased fiber–fiber interaction. The effect of fiber length between the differently produced compounds is more pronounced, with an increase between 5 and 10% for higher fiber lengths, which was to be expected. Tensile strength is measured at higher strains than the elastic modulus; therefore, higher fiber lengths show more pronounced effects as the longer fibers can carry higher loads when these are strained higher. This finding is supported by the results from the yield strain of the different composites, where we can see a reduction in strain (due to a reduction in polymer chain mobility) due to the fiber content, on the one hand, but also due to the higher carbon fiber lengths present in the composites, which were produced with less residence time in the compounder (dosing in zone 8). The trends from these findings are also in agreement with the literature for glass- [1,2,10] and carbon-fiber-reinforced composites [26,27]. Composite density increases with increasing fiber volume content, as the carbon fibers exhibit a higher density (approx. 1.7 g/cm^3^) than the PP matrix (approx. 0.91 g/cm^3^). In this case, no significant influence of the fiber length can be observed.

**Figure 5:**
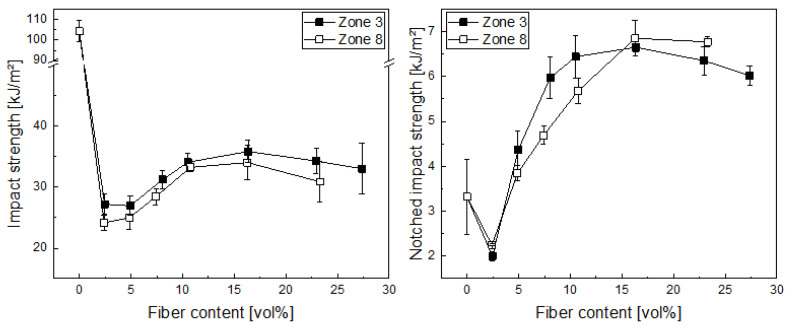
Unnotched (**left**) and notched (**right**) impact strength vs. the fiber volume content for carbon-fiber polypropylene composites produced with two different dosing positions in compounding (zone 3 and 8).

In the case of the unnotched impact strength (Figure 5), we found an initial drop due to changes in the fracture behavior from a flexible test specimen to a stiff one. Afterwards, an increase in impact strength up to about 15 vol% (which equals approx. 30 wt%) of carbon fibers can be found, which is essentially leveled out at higher fiber volume fractions. We expect this occurred due to the decrease in fiber length at higher fiber volume fractions and increased fiber–fiber interaction. The difference between the longer fibers with the reduced residence time in compounding (zone 8) and the shorter ones here is not significant, as the error bars, which indicate the standard deviation, overlap for most data points. Looking at the notched impact strength, as similar picture can be observed, although the initial decrease is much lower, as polypropylene homopolymers are known for being sensitive to notching in impact behavior. Then, the impact strength again increases up to 15 vol%, levelling out after that. Again, the differences among the different fiber lengths are very small and can be considered not significant.

## 4. Conclusions

In this work, we investigated the influence of the compounding process on the fiber length and the properties of carbon-fiber-reinforced polypropylene. We found that the dosing position has a major influence on process stability and the resulting fiber length after the compounding step. With dosing the fibers earlier in the co-rotating twin screw extruder (zone 3), the longer residence time results in a more stable process, as the fibers are homogenized better and the entrapped air is degassed, but also the residual fiber length is shorter, while dosing at a later point (zone 8) produces a less stable process, but longer fibers. The dosing itself is stable up to 30 wt% of carbon fibers (at the set throughput of 10 kg/h) and shows a deviation at higher dosages, most probably due to the light weight and high volume of the carbon fibers.

The injection-molding step decreases the fiber length (from about 900–500 µm to 500–250 µm) and homogenizes the mixtures, but although the longer fibers retained by dosing in the later zone 8 at the compounding are broken down more than the shorter ones, the longer fibers remain longer, at least at the lower fiber contents. This longer fiber length, although in absolute values only between 50 and 100 µm longer, shows increased tensile strength as well as elastic modulus as well as a reduced strain at yield. With such a small change in the process, and a subsequent gain of 50–100 µm of fiber length, the overall properties increased between 5 and 10% for the tensile properties and there was some increase in impact strength. In our opinion, this shows that even small improvements in processing can aid in improving composite properties, which then can be used to reduce the material input for different applications, which is beneficial, especially in automotive and transportation applications. Another approach to utilize this would be to reduce the fiber content by a small percentage, thus aiding the preservation of fiber length and also providing an economic advantage, as the carbon fibers are more expensive than polypropylene ones.

Looking at the composite properties overall, about 15 vol% (30 wt%) seems to be the optimum identified in our study. Here, processing works as intended and properties such as elastic modulus and tensile and impact strength are well balanced, as increasing the fiber content results in only small to no increases in the latter two, but also considerably more instabilities in the processing. Nevertheless, the longer fibers show better or at least equal performance in the investigated range, although the length difference is rather small. In our opinion, this also shows that, due to the considerable fiber length reduction, further investigation regarding injection molding should be undertaken to preserve fiber length better for an increased performance.

## Figures and Tables

**Table 1:** Composite formulations produced in this study.

CF Dosing Zone	HE125MO (PP) [wt%]	PX35-55 (CF) [wt%]	Scona TPPP 8112 GA [wt%]
3	92	5	3
	87	10	3
	82	15	3
	77	20	3
	67	30	3
	57	40	3
	47	50	3
8	92	5	3
	87	10	3
	82	15	3
	77	20	3
	67	30	3
	57	40	3
	47	50	3

**Table 2:** Temperature profile (top lines, all values given in °C) and screw geometry (bottom line, each zone represents approx. 4 L/D, except the die) of the co-rotating twin-screw extruder. The screw geometry is given as: c—conveying elements (feed screws, each 1 L/D long), kb1—kneading block 1 (consisting of 5 × 30°, 7 × 60°, 4 × 90° kneading blocks) for polymer melting, kb2—kneading block 2 (consisting of 7 × 60°, 6 × 90°) for fiber mixing, and kb3—kneading block 2 (consisting of 4 × 60°, 4 × 90°) for fiber mixing. Each kneading block element represents ¼ L/D.

Intake	Zone 2	Zone 3	Zone 4	Zone 5	Zone 6	Zone 7	Zone 8	Zone 9	Zone 10	Die
40	210	230	230	230	220	210	200	200	200	190
c	c	kb1	c	c	c	kb2	c	kb3	c	-

**Table 3:** Injection-molding parameters applied for all mixtures. Parameters were chosen following ISO 3167 and adjusted to obtain the proper mold filling. Additionally, a low back pressure was chosen to inflict less shear, and therefore less fiber damage, to the compounds.

Parameter	Set Value
Mass temperature	230 °C
Mold temperature	40 °C
Dosing volume	45 cm^3^
Injection speed	0.35 m/s
Back pressure	10 bar
Holding time	40 s
Cycle time	60 s

## Data Availability

The data presented in this study are available upon request from the corresponding author.
